# Two New Trematodes of Family Acanthocolpidae Luhe, 1906 From Marine Fish *Leiognathus daura* (Cuvier) from the Coast of Puri, Orissa, India

**Published:** 2013

**Authors:** Sushma MISHRA, Satish CHANDRA, Anand Murari SAXENA

**Affiliations:** 1 Department of Zoology, University of Lucknow, Lucknow, India

**Keywords:** Digenea, Marine fishes, Acanthocolpus, Parasite, India

## Abstract

**Background:**

Genus *Acanthocolpus* (Trematoda: Acanthocolpiidae) is one of the most important zoonotic digenean with wide geographic distribution in the world. The purpose of the present study was to describe morphological and morphometrical characteristics of *Acanthocolpus* species, currently prevalent in marine fish fauna of Puri coast, Orissa, India.

**Methods:**

Gastro-intestinal organs of *Leiognathus daura* (Cuvier) in Puri coast, Orissa, India, were examined for infectivity with digenean trematode species. For examination and measurements of helminthes, acetoalum carmine staining was performed, followed by camera Lucida drawings of morphological characters and measurements of morphometrical criteria with a calibrated microscope. Using valid trematode systematic keys, almost all the parasites were identified at the level of species.

**Results:**

Overall, 36 marine fishes were found infected with at least one species of *Acanthocolpus*. Considering morphological characteristics of *Acanthocolpus*, two species were identified as new species including *Acanthocolpus durghai* sp.nov. and *Acanthocolpus amrawatai* sp.nov.

**Conclusion:**

During the survey of helminth parasites, collected six different species of the genus *Acanthocolpus, out* of these two are new species, another is redescribed to show certain variation, the new parasite was obtain from the intestine of marine fish *Leiognathus daura* (Cuvier)

## Introduction

In this paper we are adding the knowledge of *Acanthocolpus* (1), especially of marine teleost fishes from the coast of Puri, Orissa (India). During the survey of helminth parasites, collected six different species of the genus *Acanthocolpus*, are found out of these two are new and four species, are rediscribed. The new species were found in the intestine of marine fish *Leiognathus daura* (Cuvier).

## Materials and Methods

During the examination of the marine fish specimens of the above genus were recovered from the intestine of marine fish. The specimens were collected and identified by fish books and cut open and thoroughly examined after that helminthes parasites were separated in Petri dish containing normal saline solution. The parasite were flattened with slight pressure of cover glass and fixed in A.F.A. fixative (50% alcohol, formalin and acetic acid in ratio of 100: 6: 2.5). They were stained in acetoalum carmine, differentiated in acid alcohol and dehydrated through ascending grade of alcohols. These were cleared in xylol and mounted in canada balsam or DPX. The diagrams were made with the help of camera Lucida. All the measurements in millimeters: unless otherwise stated.

## Results

### Description Family: Acanthocolpiidae Subfabily: Acanthocolpiinae Acanthocolpus durghai sp nov. (Figure-1)

Body is elongated, aspinose, slender 1.69-1.86 mm long, 0.28-0.30 mm wide. Oral sucker Reni form terminal, 0.05-0.08 mm long, 0.08-0.11 mm wide. Pre-pharynx Long, narrow, 0.04-0.11 mm long, 0.01-0.01mm wide. Pharynx small ovoid muscular 0.03-0.04 mm long 0.02-0.02 mm wide. Oesophagus absent, intestinal caeca is simple extending up to hind end of body. Ventral sucker is present at anterior extremity, sub-spherical, pre-testicular, larger than oral sucker, 0.11-0.13 mm long, 0.12 - 0.14 mm wide at 0.10-0.20 mm from anterior extremity.

**Fig. 1:**
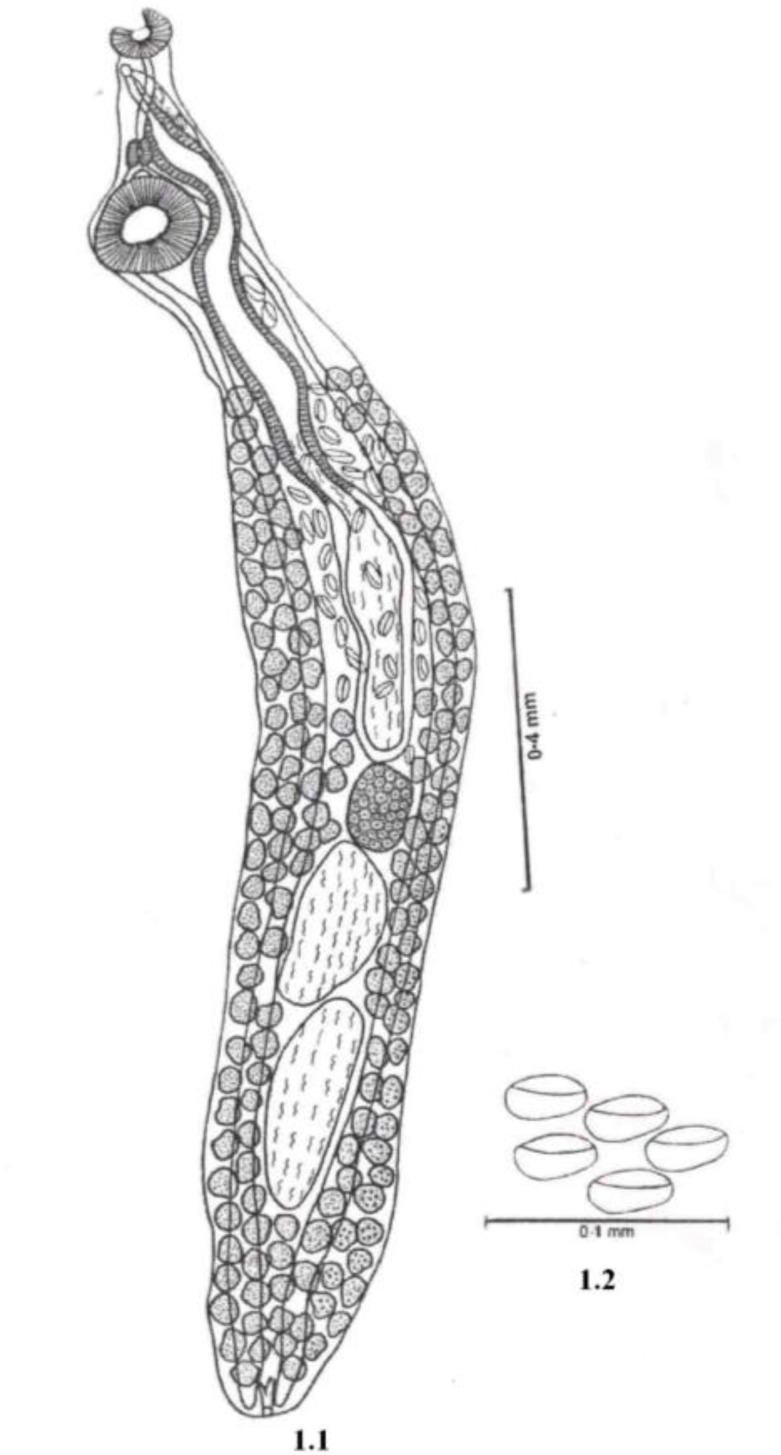
*Acanthocolpus durghai sp. nov.;* 1.1 Ventral view of adult; 1.2 Eggs

Excretory bladder ‘Y’-shaped, excretory pore terminal. Genital pore sub-median, lying on right side of body behind oral sucker at 0.05-0.13 mm from anterior extremity. Testes two, elongated, inter-caecal, in posterior third of the body. Anterior testis 0.19-0.22 mm long, 0.11 -0.13 mm wide at 0.86-1.14 mm from anterior extremity. Posterior testis 0.09-0.30 mm long, 0.10-0.12 mm wide at 1.07-1.36 mm from anterior extremity. Cirrus sac long, slender, sinuous, 0.56-1.01 mm long, 0.08-0.09 mm wide extending from just in front of ovary up to little behind to oral sucker. Vesicula seminalis elongated, 0.32-0.53 mm long, 0.06-0.07 mm wide. Pars-prostatica long, tubular, 0.25-0.49 mm long surrounded by large number of prostate gland cells.

Ovary sub-spherical, sub-median, intercaecal, pre-testicular, post-equatorial just in front of anterior testis, 0.11-0.12 mm long, 0.09-0.10 mm wide at 0.75 - 1.07 mm from anterior extremity. Vitelleria follicle extending from middle of cirrus sac up to hind end of body. Uterus pre-ovarian. Metaterm muscular. Eggs large, elongated, operculated, 0.03-0.04 mm long, 0.01-0.02 mm wide.

## Discussion

The present form belongs to genus *Acanthocolpus* Luhe, 1906 comprises of the following species *A. liodorus* (1) from Ceylon Synonym *A. luhei* (2); *A. indicum* (2) from Karachi; *A. orientalis* (2) from Karachi; *A. tenuis* (3) from Fije, *A. brasilensis* (4) from Guanabara, *A. caballeroi* (5) from Ratna Giri; *A. guptai, A. puriensis, A. lucknowensis* (6) from Puri, Orissa, *A. inglisi* (6) from Puri, Orissa, *A. srivastavai, A. valiyathurai* (7) from Trivandrum, Kerala, *A. chorinesmusi, A. equulai, A. lutijanusi* (8) from Tamilnadu and Puri, Orissa; *A. microtesticulus* (9) from Maharashtra are known so far. *A. luhei* was considered as a synonym of *A. liodorus* (10). *A. luhei* was distinguished from *A. liodorus* in the anterior extent of vitellaria, sucker ratio and in the absence of acetabular penduncle (11). Later on Gupta and Gupta (12) followed Yamaguti (10) and considered all the characters mentioned by Hafeezullah, (11) are variable characters. Author agrees with Yamaguti (10) and Gupta and Gupta (12) in considering *A. luhei* (2) as a synonym of *A. liodorus* (1).

The present form differs from all these species except *A. luhei, A. indicum, A. orientalis* and *A. chorinesmusi* in the absence of acetabular peduncle.

The present form differs from all these forms in having reniform oral sucker instead of sub-spherical or conical, genital pore lying on little posterior to oral sucker instead of just pre-acetabular region or at middle of ventral sucker, cirrus sac extending from just anterior to ovary instead of separated from uterine coils and in relative shape and size of various organ.

### Description Acanthocolpus amrawatai sp. nov. (Figure-2)

Body elongated, aspinose, slender, 1.91-3.18 mm long, 0.27-0.37 mm wide. Oral sucker sub-terminal, sub-spherical, 0.11-0.12 mm long, 0.09-0.12 mm wide. Pre-pharynx long, narrow, tubular, 0.04 – 0.13 mm long, 0.02 mm wide. Pharynx ovoid, muscular 0.08-0.09 mm long, 0.03-0.03 mm wide. Oesophagous very short. Intestinal caeca simple, extending up to near posterior extremity. Ventral sucker larger than oral sucker, sub-spherical, pedunculated, 0.11-0.15 mm long, 0.15-0.16 mm wide at 0.11-0.36 mm from anterior extremity.

**Fig. 2:**
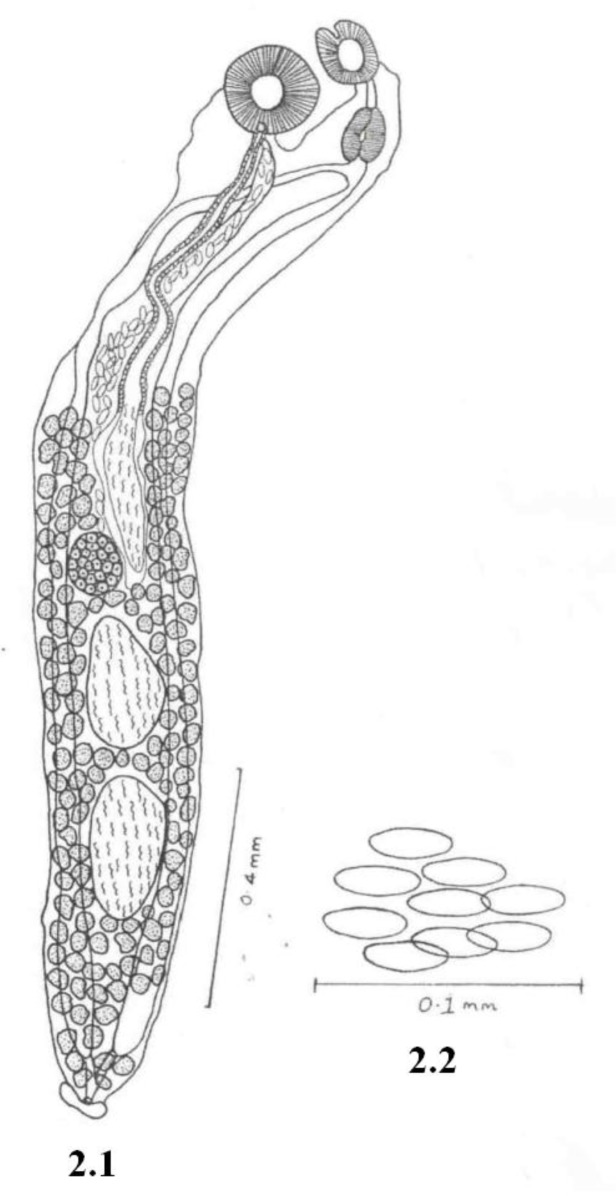
*Acanthocolpus amarawatai* sp. *nov.;* 2.1 Ventral view of adult; 2.2 Eggs

Excretory bladder ‘Y’- shaped, excretory pore terminal. Genital pore lying posterior to ventral sucker at 0.21-0.37 mm from anterior extremity. Testes two, lying one behind other, inter-caecal, post-ovarian, in posterior third of the body. Anterior testis pear shaped 0.23-0.29 mm long, 0.12-0.13 mm wide at 1.09-1.94 mm from anterior extremity. Posterior testis elongated 0.24-0.34 mm long, 0.12-0.12 mm wide at 1.37-2.33 mm from anterior extremity. Cirrus sac long, elongated, tubular, sinuous, 0.84-1.53 mm long, extending from anterio-lateral to ovary up to ventral sucker. Vesicula seminalis elongated 0.33-0.54 mm long, 0.05-0.06 mm wide. Pars-prostatica long, narrow, tubular, 0.54-0.98 mm long, surrounded by large number of prostate gland cell.

Ovary spherical, sub-median, pre-testicular, inter-caecal, little anterior to anterior testis, 0.10-0.16 mm long, 0.09-0.17 mm wide at 0.97-1.76 mm from anterior extremity. Vitellaria follicular along side of body, extending from middle of cirrus sac up to hind end of body, Uterus pre-ovarian, Metaterm muscular, short, Eggs elongated, elliptical, non-operculated, 0.03-0.04 mm long, 0.01-0.01 mm wide.

## Discussion

The present form closely resembles with *A. tenuis* (3) *, A. cabelleroi* (5), *A. inglisi* (12), *A. equualai*, *A. lutijanusi* (8), *A. arpitai*, *A. apoorvai* (13) in having well developed acetabular peduncle.

The present form differ from all they above form except *A. inguisi* and *A. cabelleroi*, *A. arpitai*, *A. apoorvai* in having body elongated slender, oral sucker terminal, sub-spherical, uterine coil not present in between ovary and testis.

However present form differs from *A. inguisi, A. cabelleroi, A. arpitai* and *A*. *apoorvai* in having genital pore lying posterior to ventral instead of at the level of pharynx, cirrus sac extending up to ventral sucker instead of level of pharynx, testes are separated from each other by vitellaria instead of lying one behind other in *A*. *inguisi*, genital pore lying anterio-ventral to ventral sucker in *A. cabelleroi*, genital pore present at level of pharynx ovary pre-equatorial instead of post equatorial, cirrus sac long instead of short, egg non-operculated instead of operculated in *A*. *arpitai* the genital pore lying on posterior to intestinal bifurcation, cirrus sac extending from just in front of ovary instead of at level of vitellaria in *A. apoorvai*, and in relative shape and size of various organs.

Thus an account of above mentioned differences as against all those described earlier, the present form may deserve the status of new species with specific name *A. amarawatai* sp.nov.
